# Structural Characterization of Hypoxia Inducible Factor α—Prolyl Hydroxylase Domain 2 Interaction through MD Simulations

**DOI:** 10.3390/ijms24054710

**Published:** 2023-03-01

**Authors:** Giorgia F. Camagni, Giovanni Minervini, Silvio C. E. Tosatto

**Affiliations:** Department of Biomedical Sciences, University of Padua, 35121 Padova, Italy

**Keywords:** molecular dynamics simulation, hypoxia, hypoxia-inducible factor, HIF-prolyl hydroxylases, VHL

## Abstract

The Prolyl Hydroxylases (PHDs) are an enzymatic family that regulates cell oxygen-sensing. PHDs hydroxylate hypoxia-inducible transcription factors α (HIFs-α) driving their proteasomal degradation. Hypoxia inhibits PHDs activity, inducing HIFs-α stabilization and cell adaptation to hypoxia. As a hallmark of cancer, hypoxia promotes neo-angiogenesis and cell proliferation. PHD isoforms are thought to have a variable impact on tumor progression. All isoforms hydroxylate HIF-α (HIF-1,2,3α) with different affinities. However, what determines these differences and how they pair with tumor growth is poorly understood. Here, molecular dynamics simulations were used to characterize the PHD2 binding properties in complexes with HIF-1α and HIF-2α. In parallel, conservation analysis and binding free energy calculations were performed to better understand PHD2 substrate affinity. Our data suggest a direct association between the PHD2 C-terminus and HIF-2α that is not observed in the PHD2/HIF-1α complex. Furthermore, our results indicate that phosphorylation of a PHD2 residue, Thr405, causes a variation in binding energy, despite the fact that this PTM has only a limited structural impact on PHD2/HIFs-α complexes. Collectively, our findings suggest that the PHD2 C-terminus may act as a molecular regulator of PHD’s activity.

## 1. Introduction

Hypoxia is a major hallmark of tumor growth. The mechanism of adaptation to hypoxia at the cellular level is mediated by the hypoxia-inducible factor (HIF) family, a transcription factor consisting of an oxygen-sensitive alpha subunit (HIF-1α and HIF-2α being the most studied isoforms) and a constitutively expressed beta subunit (HIF-β/ARNT) [1]. Its activity is regulated by Prolyl Hydroxylase Domain-Containing Proteins (PHDs) belonging to the 2-oxoglutarate-dependent dioxygenases superfamily. The enzymatic activity of these enzymes depends on a ferrous iron cofactor, a co-substrate, i.e., 2-oxoglutarate (2-OG), and molecular oxygen [2]. In normoxic conditions, PHDs hydroxylate specific proline residues (Pro402 and Pro564 in HIF-1α, Pro405, and Pro531 in HIF-2α) located in the so-called LxxLAP motifs of the HIFs-α family [3,4,5,6]. Hydroxylation allows the subsequent interaction with the von Hippel–Lindau tumor suppressor protein (pVHL), a member of the ubiquitin E3 ligase complex termed VCB, which includes Elongin-B, Elongin-C, and Cullin-2p [7,8]. This interaction leads to the ubiquitination and proteasome-mediated degradation of hypoxia-inducible factors α [9,10]. Furthermore, pVHL is the main actor in von Hippel–Lindau disease (VHL) [11], a human hereditary predisposition to develop cancer caused by mutation of the homonymous gene. Typical manifestations of the diseases include clear-cell renal cell carcinoma (ccRCC), retinal- and cerebellar-hemangioblastoma, pheochromocytoma (PCC), and pancreatic neuroendocrine tumors, in addition to pancreatic and renal cysts [12,13,14,15]. Hypoxic conditions reduce PHD activity allowing HIFs-α to escape pVHL recognition. HIFs-α accumulate in the cytosol and translocate to the nucleus, where they dimerize with HIF-1β to regulate the transcription of multiple genes involved in hypoxia adaptive response, including the vascular endothelial growth factor (VEGF), erythropoietin (EPO), pyruvate dehydrogenase kinase-1 (PDK1), and glucose transporter-1 (GLUT-1) [16,17]. Although the PHDs are mostly inhibited in hypoxia, their activity is still observed at low oxygen levels, highlighting their sensitivity to changes in oxygen availability and their role as cellular oxygen sensors [18,19]. The PHDs family consists of three canonical members: PHD1 (EglN2), PHD2 (EglN1), PHD3 (EglN3), and a more recently discovered PHD4-TM (EglN4). All share a dioxygenase domain at the C-terminal, while the N-terminal is less conserved [20]. They show specific subcellular localizations and tissue-specific expressions. Indeed, PHD1 is predominantly expressed in the nucleus, PHD2 in the cytoplasm, and PHD3 in both. All these isoforms are ubiquitously expressed in human tissues; however, PHD2 is reported to be more abundant than others [21]. PHD1 is predominantly found in the testes, brain, kidney, heart, and liver, whereas PHD3 is mostly expressed in the cardiac tissue [21,22]. All members of this enzyme family hydroxylate the conserved proline residues present in HIFs-α family members; however, they present different affinities. PHD2 appears to have a greater affinity for HIF-1α under normoxic conditions, while PHD1 and PHD3 are proposed to predominantly contribute to HIF-2α regulation [23,24,25]. The cell oxygen sensing system is altered in many tumors, which prefer glycolytic metabolism despite being in the presence of oxygen (the Warburg effect). This results in the inactivation of prolyl hydroxylases, stabilization of HIFs-α, and subsequently, angiogenesis, proliferation, and cell survival to occur. Since PHDs directly regulate HIFs-α activity, they can be considered a central regulator of tumor development. However, their behavior in the context of tumors remains controversial. It has been observed that PHD isoforms have a variable and cell-dependent impact on tumor progression. Their inhibition can either promote or inhibit tumor proliferation. For instance, PHD3 inhibits colon and gastric cancer growth while promoting it in the ccRCC and maintaining high levels of HIF-2α. Again, overexpression of PHD2 inhibits liver cancer growth. On the other hand, its inhibition reduces the growth of osteosarcoma [24,25]. What determines the pro- and anti-tumor functions of each isoform, as well as their different substrate affinities is still poorly understood. Here, we used molecular dynamics simulations to characterize the PHD2 substrate specificity in binding HIF-1α and HIF-2α by identifying specific intermolecular interactions of each substrate. We also simulated the phospho-Thr405 (TPO) located on the PHD2 C-terminus to investigate its role in the binding process. Finally, we calculated the binding free energy to better understand the substrate affinity.

## 2. Results

### 2.1. Homology Modeling

**Figure 1:**
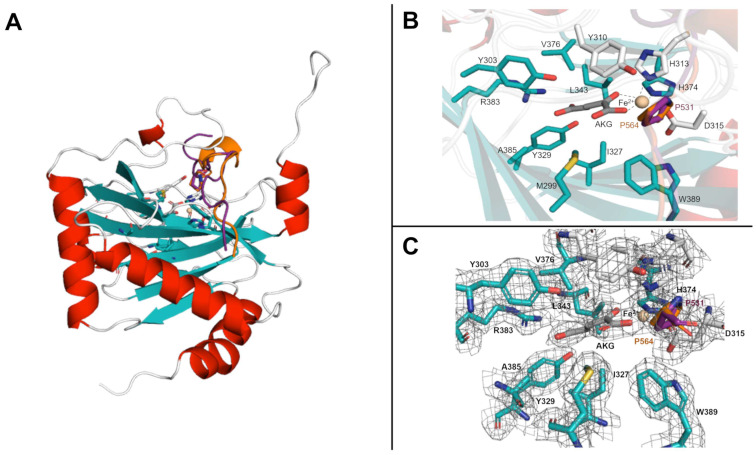
Overview of PHD2 structure. (**A**) A cartoon representation of the PHD2 enzyme in complex with its targets. The HIF-1α peptide is represented in orange, while purple is for the HIF-2α peptide. (**B**) A zoomed-in view of the PHD2 catalytic site. Key residues for substrate binding and enzymatic activity are presented as sticks. (**C**) A mesh view highlighting the PHD2 binding site in complex with the HIF-s substrates and co-substrate α-ketoglutarate (AKG).

We started our investigation by generating a tridimensional structure of the PHD2/HIF-2α complex by homology modeling. To this end, the PHD2/HIF-1α complex crystallized structure (PDB ID: 6YW3) has been used as a template. The experimental structure of HIF-1α in complex with PHD2 covers 17 amino acids (558–574), a linear motif corresponding to its C-terminal oxygen-dependent degradation domain (CODD), and also includes the conserved LxxLAP sequence motif, where the P indicates the hydroxyl acceptor proline (Figure 1).

**Figure 2:**
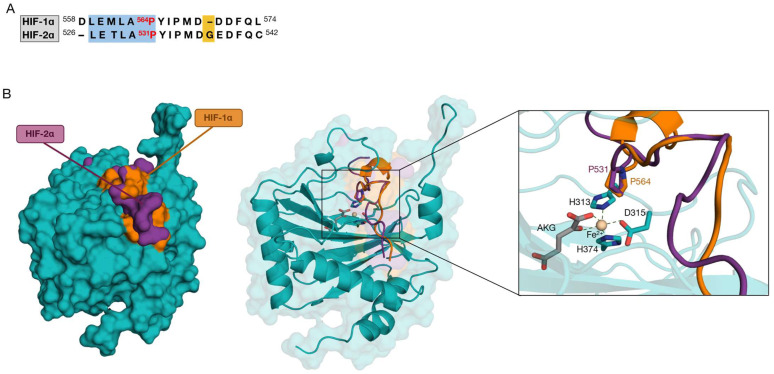
Sequence alignment and homology modeling of HIF-2α. (**A**) Sequence alignment between the 17 amino acid residues of HIF-1α (558–574) and HIF-2α (526–542). In red, the hydroxyl acceptor proline. LxxLAP motifs are shown in the light blue box. In yellow, the presence of glycine in HIF-2α leads to the formation of a gap in the HIF-1α sequence. (**B**) The **left** image shows the surfaces of the PHD2 complex (teal), with the HIF-1α (orange)/HIF-2α (purple) superposed. The **right** box shows the catalytic site and the conserved HIF-2α Pro531 position.

As there is insufficient data to indicate how HIF-2α arranges itself in the enzymatic binding pocket, we hypothesize that the position of the proline subjected to hydroxylation (HIF-1α Pro564 and HIF-2α Pro531) should be preserved. The alignment and the resulting model are shown in Figure 2.

### 2.2. Molecular Dynamics Simulations and Interaction Analysis

#### 2.2.1. PHD2/HIF-1α and PHD2/HIF-2α Complexes

**Figure 3:**
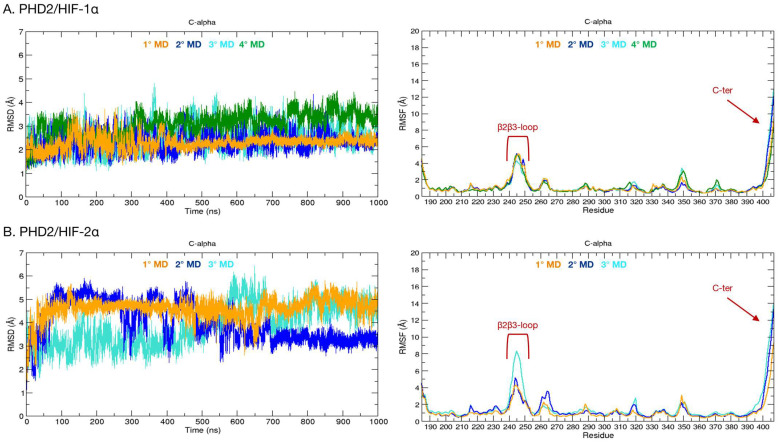
RMSD and RMSF plots calculated on PHD2/HIF-1α and PHD2/HIF-2 α complexes C-alpha over time. (**A**) RMSD (**left**) and RMSF (**right**) of the PHD2/HIF-1α complex. The four simulations that were run are shown in different colors: the first (orange), the second (blue), the third (turquoise), and the fourth (green). (**B**) RMSD (**left**) and RMSF (**right**) plots of the PHD2/HIF-2α complex. The first simulation is indicated in orange, the second in blue, and the third in turquoise.

**Figure 4:**
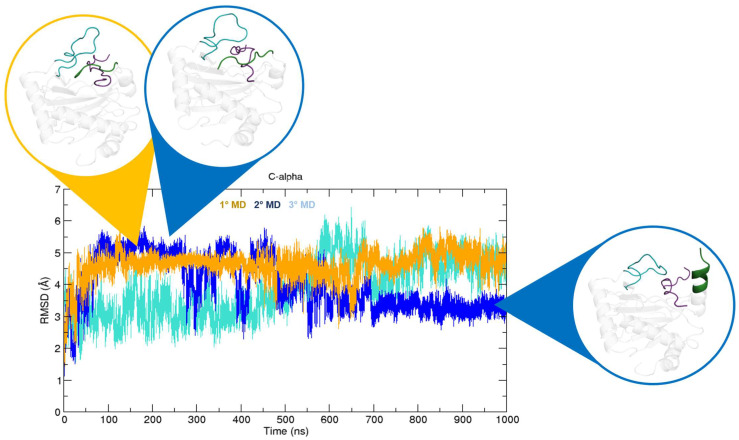
PHD2 C-terminus conformations. The C-terminus interacts with HIF-2α during the entire first run (orange), and in the second run (blue) between 100 and 250 ns. Further, the C-terminus appears to assume a mainly alpha secondary structure during the last 300 ns of the second run (blue).

The crystal structure of PHD2 is composed of the domain containing the catalytic site (185–407) and two disordered regions, i.e., the β2β3-loop (237–254), which is involved in the binding process, and the C-terminus (400–407). In order to characterize the substrate specificity of this isoform and identify specific inter-molecule interactions, 1 µs long molecular dynamics (MD) simulations of the complexes formed by PHD2 and HIF-1α/HIF-2α were carried out (Figure 3). The root-mean-square deviation (RMSD) and root-mean-square fluctuation (RMSF) plots of the PHD2/HIF-1α complex indicate that the systems remain stable for the entire simulation time with only moderate fluctuations (Figure 3A). The regions showing the greatest fluctuations are located at residues 237–254 and at residues 400–407, corresponding to the β2β3-loop and the C-terminus, respectively (Figure 3A). Differently, the PHD2/HIF-2α complex shows an important entropic effect in all simulation runs, with RMSD values reaching ~6 Å (Figure 3B). As shown in Figure 3B, regions with significant fluctuations are consistently observed in the β2β3-loop and C-terminal regions. Such behavior in both systems is expected, as these are disordered regions characterized by a high degree of conformational freedom. In detail, in the first simulation (orange), the complex is always stable and assumes a conformation in which the C-terminal region interacts with HIF-2α (Figure 4). The same conformation is observed in the second simulation run (blue) between 100 and 250 ns. Moreover, the system is very stable even in the last 300 ns of simulation, in which the PHD2 C-terminus appears to assume a mainly alpha secondary structure (Figure 4).

Based on these findings, we hypothesized that the C-terminus may play a role in the binding process by stabilizing the substrate in the active site. Indeed, we observed a RMSD shift depending on the conformation assumed by the C-terminus, with the value reaching 5 Å when the C-terminus directly interacts with HIF-2α. In contrast, a lower RMSD value of 3.5 Å is registered when the C-terminus assumes a mainly alpha conformation. These findings suggest that the binding site closing/opening due to C-terminus steric hindrance may increase the energy content of the complex. A certain degree of instability, however, was also registered in the third replica, where the C-terminus assumed multiple conformations during the entire simulation time. At 580 ns in particular, we observed a sudden spike in the RMSD value that is promoted by the breaking of van der Waals (VDW) interactions between the PHD2 residue Trp258 and HIF-1α Pro534. In order to investigate the molecular details behind the substrate specificity reported for PHDs, the specific and non-specific intermolecular interactions of PHD2 in complex with HIF-1α and HIF-2α were also analyzed. Both of these substrates consist of 17 amino acids, spanning residues 558–574 and 526–542 for HIF-1α and HIF-2α, respectively (Figure 2A). Our analysis shows that two bonds formed by Asp536-Arg396 and Glu538-Arg396 are relevant in stabilizing HIF-2α in the binding pocket. The same Arg also interacts with Pro567 of HIF-1α; however, this interaction appears not to be shared among replicas. Furthermore, HIF-1α forms two very stable H-bonds with PHD2; Leu562-Tyr310 and Pro564-Arg322. In both systems, Lys297 residue seems to play a role in stabilizing the substrate, especially in the complex containing HIF-2α. Based on our simulations, PHD2 Lys297 forms ionic bonds with HIF-2α residues Asp536, Glu538, and Asp539. Similarly, Lys297 engages electrostatic interactions with Asp570 and Asp569 when complexed with HIF-1α. The role of these negatively charged residues in driving the binding with PHD2 is also supported by their conservation (Figure 2A). In the PHD2/HIF-1α complex, the ionic bonds that Asp571 establishes with Arg396 and Lys400 are therefore more relevant. In two simulation runs of the PHD2/HIF-2α complex, we observed a single ionic bond that seemed to stabilize the direct interaction between the PHD2 C-terminus and the substrate. In particular, Lys402 residue interacts with Asp536 throughout the entire first simulation, while in the second simulation run, Lys402 interacts with Asp539 for ~150 ns. Contrarily, the ionic bond formed by the pair Glu538-Lys262, observed only in the last 300 ns of the second simulation run, suggests that this specific interaction is essential to stabilize a complex conformation, prompting the PHD2 C-terminus to increase its secondary structure content (Figure 4). Multiple constant VDW interactions were also identified in all simulations. Among them, we find that Phe391 interacts with Met535 of HIF-2α and Asp571 of HIF-1α. The same isoleucine (namely Ile533 in HIF-2α and Ile566 in HIF-1α) interacts with Arg322 and Thr296. All these interactions are more frequently observed in the HIF-1α complex than in the HIF-2α complex, where the binding to Trp389 is more stable than the others. The Trp258 residue binding Pro534 of HIF-2α and Pro567 of HIF-1α turns out to be an important interaction for both substrates. These two interactions remain stable for the entire simulation time and are shared among all replicas. Their breaking is also decisive in driving the conformational change occurring at 580 ns in the third replica of the PHD2/HIF-2α complex. A conserved phenylalanine residue of HIF-1α and HIF-2α (i.e., Phe572 and Phe540, respectively), establishes multiple VDW interactions relevant for the stabilization of both substrates in the catalytic pocket. Interestingly, it interacts with different residues depending on the substrate involved, as its side chain assumes an opposite conformation in the two complexes. In particular, it interacts with Arg295 in the PHD2/HIF-1α, while with PHD2/HIF-2α it interacts with residues forming the fourth PHD2 α-helix, i.e., Arg396, Ala399, and Lys400. Additionally, Leu574, the last residue included in the HIF-1α CODD, forms stable VDW interactions with Asp277, Ile280, and Asn293. However, due to a difference in the amino acid sequence, these bonds are not observed in the complex with HIF-2α (Figure 2A). Similarly, the pair, Ala563-Pro317, is also PHD2/HIF-1α complex-specific. A major difference between the two substrates concerns the interaction involving the proline residue targeted for hydroxylation. Indeed, the HIF-1α Pro564 forms a stable VDW with His313, an amino acid belonging to the catalytic triad in iron coordination, highlighting its importance in maintaining the catalytic site. Instead, the HIF-2α Pro531 is stabilized by interacting with the β2β3-loop, particularly with Val241. It also interacts with His313; however, this specific contact appears to be less stable and was observed in just one simulation. These findings explain why, although the HIF-2α N-terminus appears to have a higher conformational flexibility, the proline residue is kept in the binding pocket in the correct position to be hydroxylated. Finally, HIF-2α Phe540 forms a π-π stack with PHD2 Tyr403. This interaction is shared among replicas, suggesting that it is relevant to stabilizing the complex. A similar π-π stack is also observed in simulations of the PHD2/HIF-1α complex (i.e., the pair Tyr565-Trp258), however its frequency is lower and inconstant among runs. All interactions are listed in Table 1. 

#### 2.2.2. Phosphorylation of PHD2-Thr405

**Figure 5:**
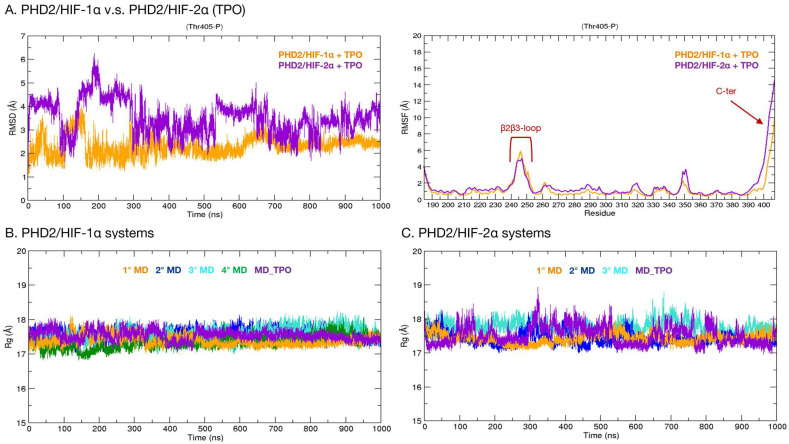
Comparison of RMSD, RMSF, and Rg plots of PHD2/HIFs-a complexes with Thr405-P (TPO). (**A**) PHD2/HIF-1α + TPO (orange) and PHD2/HIF-2α + TPO (purple). (**B**) Comparison of the gyration radius between all systems of the PHD2/HIF-1α complex: first simulation (orange), second run (blue), third (turquoise), fourth (green), and simulation with phosphorylation (purple). (**C**) Comparison of gyration radii among all systems of the PHD2/HIF-2α complex: first simulation (orange), second simulation (blue), third simulation (turquoise), and simulation with phosphorylation (purple).

Considering our results, which indicate the PHD2 C-terminus as a putative relevant actor in modulating PHD2 substrate specificity, we wondered whether post-translational modification of residues in this region may activate a further layer of regulation. The PhosphoSitePlus [26] (www.phosphosite.org, accessed on 15 August 2022) database reports that Thr405 located in the C-terminus is a phosphorylation site found in leukemia cells. To investigate if this modification may have an impact on the PHD2/HIFs-α binding, we simulated the phosphorylation of Thr405 (TPO) and evaluated its impact on the complexes. The phosphorylated PHD2/HIF-1α system exhibits a general trajectory dynamic resembling data obtained from the unmodified complex, also sharing a comparable degree of flexibility for both the disordered regions forming the β2β3-loop and the C-terminus (Figure 5A). This finding suggests that phospho-Thr405 does not affect the general stability of the complex, as also indicated by the radius of gyration (Rg) profile, which remains stable in the range of 17–18 Å in all simulations. Similarly, the modified PHD2 complexed with HIF-2α shows a dynamic behavior comparable to that obtained from other simulations without phosphorylation. Indeed, RMSD oscillations are similar, mainly due to the pronounced flexibility of the β2β3-loop and C-terminus (Figure 5A). Measurement of Rg variation also showed a substantial conservation of secondary structure; we then excluded any possible local or long-distance influence of the phosphorylated C-terminus on the PHD2 structure. Additionally, in these runs, the PHD C-terminus is the protein region showing the greatest conformational freedom, which influences protein stability. Its phosphorylation state, however, appears to have only a modest or null effect on PHD2/HIFs-α.

**Figure 6:**
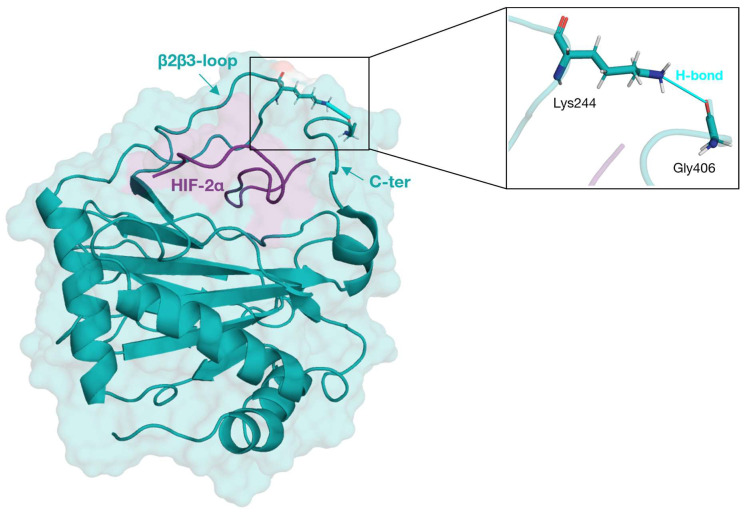
β2β3-loop and C-terminus H-bond interaction. PHD2 (teal) and HIF-2α (purple) are represented in cartoon style. Lys244 and Gly406 residues are represented by licorice and H-bonds (turquoise) by a stick.

Interestingly, we observed some change in the residue-residue interactions, indicating that phopho-Thr405 may retain a possible regulative role in modulating PHD2 substrate specificity. Our simulations also show that Hys313 within the PHD2 catalytic triad binds to Ala530 with a more stable interaction than that observed in systems without phosphorylation. Furthermore, during the entire simulation time, the direct interaction of the C-terminal with HIF-2α is apparently lost, with HIF-2α engaging in a novel interaction with the β2β3-loop via a H-bond formed by the pair Gly406-Lys244 (Figure 6). Although this bond does not persist for the whole simulation, it suggests that when PHD2 interacts with HIF-2α, the C-terminus may assume a dynamic behavior, leading it to close the binding pocket. This data agrees with the idea of a PHD2 C-terminus involved in modulating PHD2 substrate specificity.

### 2.3. Cluster Analysis

We then performed a RMSD-based structural hierarchical and state clustering analysis to extract central conformational states from all the MD simulation trajectories (Table 2). As expected, the conformation analysis shows that PHD2 tends to explore fewer conformations when it is in complex with HIF-1α, i.e., an experimental 3D structure, indicating a higher stability of this system with respect to the PHD2/HIF-2α complex (obtained from molecular modeling). Phosphorylation of Thr405 (TPO) seems to further stabilize the PHD2/HIF-1α as the number of clusters recapitulating the entire trajectory of this system is lower than the average of those with no TPO. In contrast, TPO seems not to introduce considerable conformational changes in the PHD2/HIF-2α complex. 

**Figure 7:**
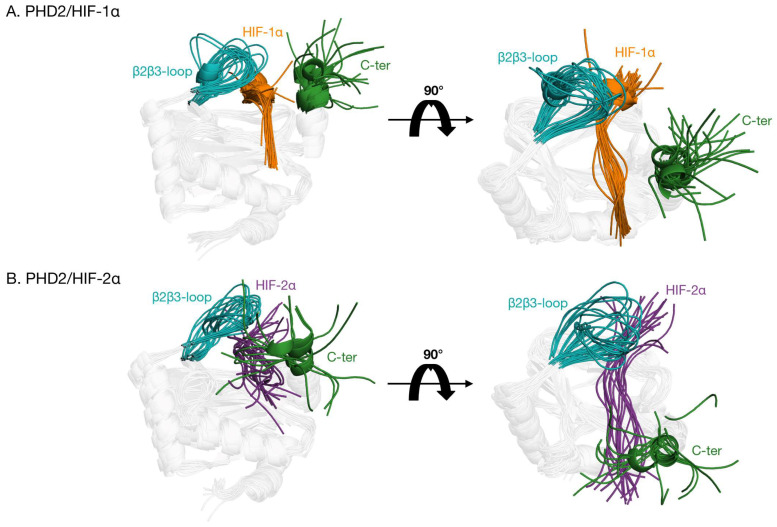
Superimpositions of the most representative PHD2/HIFs-α complexes conformations, with the β2β3-loop colored in teal, C-terminal in green, HIF-1α in orange, and HIF-2α in purple. On the left, a bird’s-eye view obtained with a 90° rotation of the complexes helps visualize the binding pocket. (**A**) PHD2/HIF-1α. (**B**) PHD2/HIF-2α.

Then we selected the first six most populated clusters for each trajectory and extracted the most representative conformations (Figure 7). The regions showing significant conformational change in both complexes are the β2β3-loop and C-terminus. This flexibility can be interpreted as a consequence of the disorder content in these specific portions of PHD2 and is in agreement with what is observed with the RMSD and RMSF inspections. Significant changes are also observed at the substrate level, in particular for the PHD2/HIF-2α complex, where one representative conformation describes the direct interaction between the protein C-terminus and the substrate, lending support to its presumed role in substrate discrimination. 

### 2.4. Binding Free Energy Analysis

**Figure 8:**
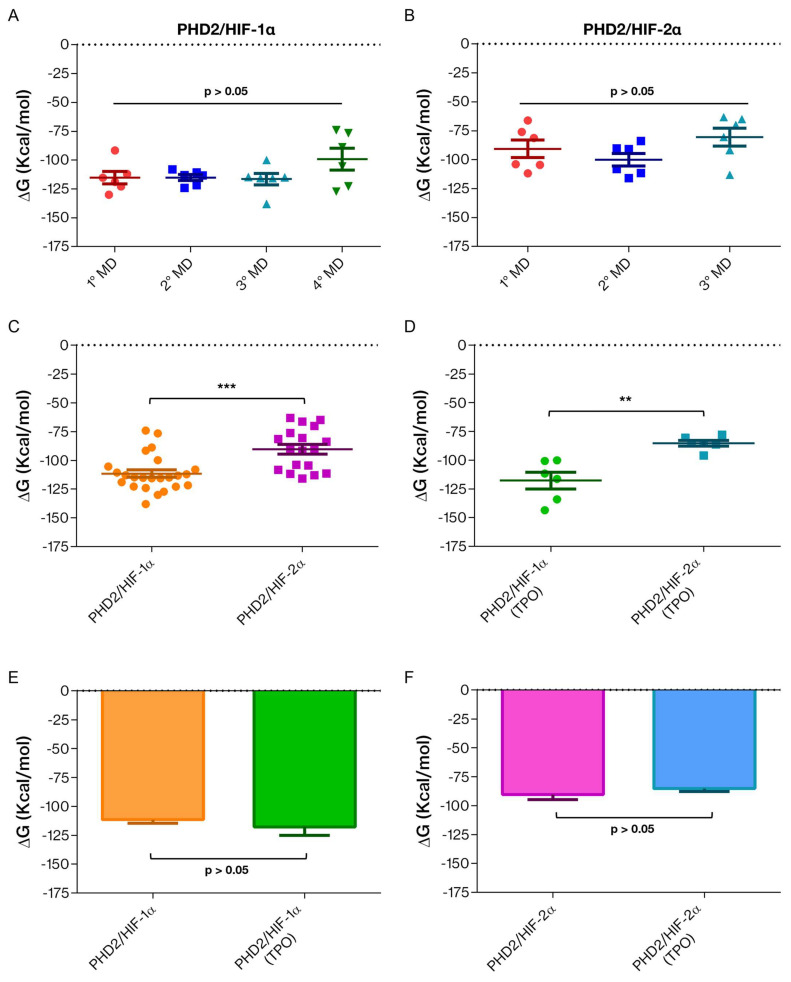
Scatter dot plots and bar plots (mean with SEM) representing the difference in the mean value of the binding free energy (ΔG) among all the complexes. (**A**) A scatter dot plot for ΔG value among the four replicas of the PHD2/HIF-1α complex, *N* = 24; 1st MD (−115.1 ± 5.356); 2nd MD (−115.1 ± 2.603); 3rd MD (−116.5 ± 4.979); and 4th MD (−99.15 ± 9.370). (**B**) Scatter dot plot for ΔG value among the three replicas of the PHD2/HIF-2α complex, *N* = 18; 1st MD (−90.64 ± 7.545); 2nd MD (−100.1 ± 5.448); and 3rd MD (−80.55 ± 7.821). (**C**) A scatter dot plot of the ΔG value shows −111.5 ± 3.204, *N* = 24 in PHD2/HIF-1α and −90.43 ± 4.270, *N* = 18 in PHD2/HIF-2α complexes. *** = *p*-value ≤ 0.001 (**D**) A scatter dot plot for the ΔG value shows −117.7 ± 7.250, N = 6 in PHD2/HIF-1α (TPO) and −85.25 ± 2.537, *N* = 6 in PHD2/HIF-2α (TPO) complexes. ** = *p*-value ≤ 0.01 (**E**) A bar plot for ΔG value −111.5 ± 3.204, *N* = 24 in PHD2/HIF-1α and −117.7 ± 7.250, *N* = 6 in PHD2/HIF-1α (TPO) complexes. (**F**) A bar plot for ΔG values −90.43 ± 4.270, *N* = 18 in PHD2/HIF-2α and −85.25 ± 2.537, *N* = 6 in PHD2/HIF-2α (TPO) complexes.

Our results showed that in the PHD2/HIF-2α complex, the C-terminus tends to “close” the binding pocket by forming an interaction with the substrate and the β2β3-loop. This behavior, which is not observed with HIF-1α, could indicate a difference in PHD2 substrate specificity. To deepen this observation, we performed binding-free energy calculations on the representative conformations obtained from the six most populated clusters (Table S1). Systems with HIF-1α present higher values of negative binding energy than those with HIF-2α, regardless of TPO, thus indicating a greater affinity of PHD2 for HIF-1α, as also reported in the literature. Interestingly, we observed a remarkable increase in the ΔG value for those conformations showing a C-terminus that closes the binding pocket by interacting with either HIF-2α or the ꞵ2ꞵ3-loop. These findings suggest that these interactions destabilize the complex differently from our initial hypothesis. Similarly, we found that Thr405 phosphorylation causes a variation in the binding free energy despite having a limited structural impact on PHD2/HIFs-α complexes. To discriminate whether the observed differences in ΔG were significant, we performed a *t*-test. Figure 8 shows the *p*-values obtained by comparing all the complexes. In particular, it was observed that there is no statistically significant difference (*p* > 0.05) between the average binding energy among replicas of the same complex (Figure 8A,B). This finding assumes a particularly important value for the PHD2/HIF-2α complex, as it suggests that despite being a model and presumably having inherent variability, its dynamic behavior remains consistent across all simulations. A statistically significant difference (*p*-value < 0.05) was found comparing PHD2/HIF-1α and PHD2/HIF-2α complexes (Figure 8C), indicating a different substrate specificity. A *p*-value < 0.05 was also found comparing the phosphorylated PHD2/HIF-1α and PHD2/HIF-2α complexes (Figure 8D). However, between the same phosphorylated and non-phosphorylated complex, a *p*-value > 0.05 was found (Figure 8E,F). We speculate that the significant difference observed among the phosphorylated complexes is due to PHD2 substrate specificity and not induced by the presence of the phosphorylation itself.

## 3. Discussion

In this work, we investigated the substrate specificity of the PHD2 enzyme in complex with the transcription factors HIF-1α and HIF-2α. PHD2 is a well-known trigger of the adaptive hypoxic response, and its enzymatic deregulation is linked to multiple human diseases, such as polycythemia and cancer [27]. This enzyme presents a different substrate specificity; however, the molecular details of this behavior are still poorly understood. Our investigations showed that residue-residue interactions between PHD2/HIF-1α and PHD2/HIF-2α are mostly conserved; however, they also suggest that the PHD2 C-terminus may play a role in favoring the interaction with specific substrates. Further reinforcing this proposed role, we report a direct interaction of the PHD2 C-terminus with HIF-2α that is not observed when the protein is in complex with HIF-1α. We also inspected the possible effect induced by the phosphorylation of Thr405, described in leukemia cells and localizing on the PHD2 C-terminal tail. Molecular dynamics simulations showed no significant difference in the stabilities of complexes upon phosphorylation; rather, we observed that the phosphorylated C-terminus engages in direct interaction with the PHD2 β2β3-loop. Although this interaction is not stably maintained for the entire simulation, it indicates a C-terminus tendency to close the binding pocket by interacting with the β2β3-loop and possibly acting as a molecular switch to activate/inactivate the enzyme. A similar tendency to close the binding pocket was also registered for the PHD2/HIF-2α complex. Binding energy calculations indicate that this closure of the active site by the PHD2 C-terminus increases the energy value, indicating that this specific interaction may reduce the substrate affinity by destabilizing the complex. We also observed a significant difference in the binding energy between the PHD2/HIF-1α and PHD2/HIF-2α complexes. Although a certain degree of variability is linked with the final conformation assumed by the complexes during simulations, particularly the C-terminus tendency to form an extra α-helix, we believe that the differences in binding energy between the two substrates may be due to an important entropic effect in the complexes formed by HIF-2α. 

## 4. Materials and Methods

### 4.1. Homology Modeling

The homology model of HIF-2α was performed using Modeller [28], since its X-ray structure exists in the hydroxylated form in complexes with the pVHL-Elongin C–Elongin B (VCB) complex (PDB ID: 6I7R). Therefore, we used the crystal structure of HIF-1α CODD (556–574) in complex with PHD2 (PDB ID: 6YW3) as a template. T-Coffee [29] was used to align the sequences. Ten models were generated, and the best-scoring one was selected using the DOPE score and subsequently modeled on HIF-1α into the PHD2 binding pocket. In order to avoid steric clashes among residues, a minimization run followed by 2 ns of NVT simulations/ensembles were performed.

### 4.2. Molecular Dynamics Simulations

The crystal structure of PHD2 in closed conformation (PDB ID: 6YW3) was used as the starting structure. For all systems simulated, N-oxalylglycine (NOG) inhibitor was replaced with α-ketoglutarate (AKG), the natural co-substrate, by superimposing the crystal structure of PHD2 (PDB ID: 6YW1) in complex with it. The manganese (II) ion (Mn^+2^) in the native structure was modified to ferrous ion (Fe^+2^). All MD simulations were performed with GROMACS (2020.6) [30], using the CHARMM36 force field [31]. The AKG parameters are not included in the force field, so we generated the corresponding parameters with CHARMM-GUI [32] and CGenFF [33], respectively, and implemented them in the CHARMM36m force field. As AKG contains two carboxyl groups that are deprotonated at pH 7, we recalculated the partial atomic charges using those of the amino acids glutamate and glutamine inserted in the force field as references and charges obtained with MOPAC [34] (Figure S1). A cubic box with a distance of 10 Å was generated, filled with TIP3P water molecules, and ionized with 0.15 M NaCl. The system was minimized using a steepest descent algorithm followed by 2 ns of NVT ensemble, 2 ns of NPT ensemble, and then by 1 µs of classical molecular dynamics (MD) simulation. The temperature was coupled with a V-rescale thermostat and maintained at 300 K, while in the NPT simulation, a Berendsen barostat was used. The pressure was maintained at 1 atm. Three independent replicas were obtained for all the systems, while a fourth replica was specifically calculated for the PHD2/HIF-1α complex. This further simulation and the following statistical analysis were considered necessary to strengthen our results. Indeed, the first simulation of this specific system presented some outliers, likely due to this trajectory being obtained from an extension up to 1 µs of an initial 500 ns run. The RMSD, RMSF, and radius of gyration were calculated with GROMACS, and plots were generated using Grace [35]. 

### 4.3. Phosphorylation of PHD2-Thr405

Information on the phosphorylation of the Threonine405 residue located on the PHD2 C-terminus was retrieved by PhosphoSitePlus [26]. This post-translational modification (PTM) was characterized in vivo by mass spectrometry. The phosphate group was added to the Threonine405 residue using UCSF Chimera’s Build structure tool [36]. A 200 ns molecular dynamics simulation was then performed to further stabilize the system. The last frame of the resulting simulation was used as the starting conformation to run a 1 µs molecular dynamics simulation.

### 4.4. Cluster Analysis

The cluster analysis was performed for all simulations using the RING PyMol plugin [37], on a local Linux workstation. Clusters were generated on Cα by imposing a RMSD threshold of 3 Å. For each system, the first 6 most representative clusters were selected to obtain an equal and comparable number. Subsequently, the most representative conformation of each cluster was used to calculate the binding free energy (see Section 4.5).

### 4.5. Binding Free Energy and the T-Test

Binding free energies were calculated using MM/GBSA free energy decomposition methods integrated into HawkDock [38], a web server that predicts and analyzes protein-protein interactions. The most representative conformation of each cluster was used to carry out the analysis. Calculations were repeated for all 6 conformations derived from each simulation, and the average of the resulting binding energy values was calculated (Table S1). A *t*-test was used to test whether the means of two populations were significantly different. In particular, a two-sample *t*-test was performed for groups with homogeneous variances and a Welch’s *t*-test or an unequal variances *t*-test for those with unequal variances (Tables S3 and S4). A Shapiro–Wilk test preceded this analysis to verify the assumption that each sample size was normally distributed (Table S2).

## 5. Conclusions

Our data, although generated in silico, confirm a greater affinity for HIF-1α than HIF-2α for PHD2. They also suggest that the PHD2 C-terminus could act as a molecular regulator of the enzyme activity. Furthermore, this investigation highlights specific residues that allow PHD2 to discriminate between HIF-1α and HIF-2α. Considering the PHDs’ role in human diseases, the identification of these sites may be of relevance for cancer research as their mutations can interfere with the binding of a specific substrate without impacting the PHD2 enzymatic activity.

## Figures and Tables

**Table 1:** Conserved and specific interactions of PHD2/HIF-1α and PHD2/HIF-2α complexes with a frequency cutoff < 20%. Gray is for conserved interactions, while HIF-1α specific interactions are marked in orange. HIF-2α specific interactions are highlighted in purple.

PHD2 Residue	HIF-1α Residue	HIF-2α Residue	Interaction Type		

Arg396	Pro567	Asp536 Glu538	H-Bond	Run 1°–2°	
Tyr310	Leu562		H-Bond		
Arg322	Pro564		H-Bond		
Lys297	Asp569 Asp570	Asp536 Glu538 Asp539	Ionic Bond		
Arg396 Lys400	Asp571	Asp536	Ionic Bond		Run 3°
Lys402		Asp536 Asp539	Ionic Bond	Run 1°–2° (C-ter closed conformation)	
Lys262		Glu538	Ionic Bond	Run 2°–3°	
Phe391	Asp571	Met535	VDW		Run 1°–3°
Thr296 Arg322 Trp389	Ile566	Ile533	VDW		
Trp258	Pro567	Pro534	VDW		
Arg295	Phe572		VDW		
Arg396 Ala399 Lys400		Phe540	VDW		
Lys404		Phe540	VDW	Run 1° (C-ter closed conformation)	
Val401		Phe540	VDW	Run 2° (C-ter closed conformation)	
Gln239 Leu240 Val241	Leu562 Ala563 Tyr565	Leu529 Ala530 Tyr532	VDW		
Asp277 Ile280 Asn293	Leu574		VDW		
Pro317	Ala563	Ala530	VDW		Run 1°–2°
His313	Pro564	Pro531	VDW		Run 2°
Val241	Pro564	Pro531	VDW	Run 1°–2°	
Tyr403		Phe540	π-π stack	Run 1°–2°	
Trp258	Tyr565	Tyr532	π-π stack	Run 2°	Run 3°

**Table 2:** Total cluster numbers of PHD2/HIF-1α, PHD2/HIF-2α, PHD2/HIF-2α with Thr405-P (TPO), and PHD2/HIF-2α with Thr405-P (TPO).

MD Simulations	PHD2/HIF-1α	PHD2/HIF-2α	PHD2/HIF-1α (TPO)	PHD2/HIF-2α (TPO)
1°	9	25	12	43
2°	25	30		
3°	19	73		
4°	15			

## Data Availability

The datasets generated during and/or analyzed during the current study are available from the corresponding author on reasonable request.

## Supplementary Materials

The following supporting information can be downloaded at: https://www.mdpi.com/article/10.3390/ijms24054710/s1.
