# Supplementary material for: Structural Characterization of Hypoxia Inducible Factor α—Prolyl Hydroxylase Domain 2 Interaction through MD Simulations
Source: Int J Mol Sci. 2023 Mar 1;24(5):4710. doi: 10.3390/ijms24054710 (PMC10003257; doi:10.3390/ijms24054710)
Supplement: Supplementary file 1 [file ijms-24-04710-s001.zip › Figure S1.pdf]

AKG

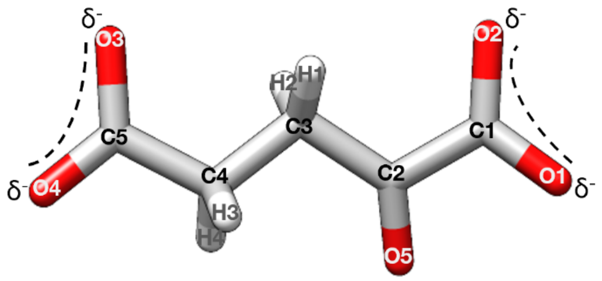

NET ATOMIC CHARGES

ATOM NO.	TYPE	CHARGE
1	C(HETATM 3601 C1 AKG A 502)	0.660045
2	O(HETATM 3602 O1 AKG A 502)	-0.758444
3	O(HETATM 3603 O2 AKG A 502)	-0.760385
4	C(HETATM 3604 C2 AKG A 502)	0.401239
5	O(HETATM 3605 O5 AKG A 502)	-0.557847
6	C(HETATM 3606 C3 AKG A 502)	-0.350598
7	C(HETATM 3607 C4 AKG A 502)	-0.379938
8	C(HETATM 3608 C5 AKG A 502)	0.740202
9	O(HETATM 3609 O3 AKG A 502)	-0.788656
10	O(HETATM 3610 O4 AKG A 502)	-0.825062
11	H(HETATM 3611 H1 AKG A 502)	0.157930
12	H(HETATM 3612 H2 AKG A 502)	0.182225
13	H(HETATM 3613 H3 AKG A 502)	0.136319
14	H(HETATM 3614 H4 AKG A 502)	0.142972

NET CHARGE ON RESIDUES

Residue	Charge	Anion or Cation?
AKG A 502	-2.000	ANION

Figure S1. Molecular structure and atomic charges of α-ketoglutarate (AKG). In the molecular structure (left) carbon atoms are indicated in grey, oxygens in red, and hydrogens in white. On the right, the net atomic charges calculated with MOPAC.
